# Gut microbiota composition is altered in postural orthostatic tachycardia syndrome and post-acute COVID-19 syndrome

**DOI:** 10.1038/s41598-024-53784-9

**Published:** 2024-02-09

**Authors:** Viktor Hamrefors, Fredrik Kahn, Madlene Holmqvist, Katherine Carlson, Roosa Varjus, Alexander Gudjonsson, Artur Fedorowski, Bodil Ohlsson

**Affiliations:** 1 Department of Clinical Sciences, Lund University, Malmö, Sweden; 2 Department of Cardiology, Skåne University Hospital, Malmö, Sweden; 3 Department of Infection Medicine, Skåne University Hospital, Malmö, Sweden; 4 Department of Clinical Sciences, Lund University, Lund, Sweden; 5 Clinical Microbiomics, Symbion, Copenhagen, Denmark; 6 Department of Cardiology, Karolinska University Hospital, Stockholm, Sweden; 7 Department of Medicine, Karolinska Institute, Stockholm, Sweden; 8 Department of Internal Medicine, Skåne University Hospital, Malmö, Sweden

**Keywords:** Functional profiling, Gut microbiota, Long COVID, Post-acute COVID-19 syndrome (PACS), Postural orthostatic tachycardia syndrome (POTS), Taxonomic profiling, Viral infection, Microbiota

## Abstract

Postural Orthostatic Tachycardia Syndrome (POTS) reflects an autonomic dysfunction, which can occur as a complication to COVID-19. Our aim was to examine gastrointestinal symptoms and gut microbiota composition in patients with POTS and post-acute COVID-19 syndrome (PACS), compared with controls. POTS patients (*n* = 27), PACS patients (*n* = 32) and controls (*n* = 39) delivered fecal samples and completed a 4-day food diary, irritable bowel syndrome-severity scoring system (IBS-SSS), and visual analog scale for IBS (VAS-IBS). A total of 98 DNA aliquots were sequenced to an average depth of 28.3 million (M) read pairs (Illumina 2 × 150 PE) per sample. Diversity and taxonomic levels of the microbiome, as well as functional abundances were calculated for POTS and PACS groups, then compared with controls. There were several differences in taxonomic composition between POTS and controls, whereas only the abundance of *Ascomycota* and *Firmicutes* differed between PACS and controls. The clinical variables total IBS-SSS, fatigue, and bloating and flatulence significantly correlated with multiple individual taxa abundances, alpha diversity, and functional abundances. We conclude that POTS, and to a less extent PACS, are associated with differences in gut microbiota composition in diversity and at several taxonomic levels. Clinical symptoms are correlated with both alpha diversity and taxonomic and functional abundances.

## Introduction

Postural orthostatic tachycardia syndrome (POTS) is one of the most common forms of cardiovascular dysautonomia that predominantly affects women in reproductive age^1^. The syndrome is characterized by abnormal heart rate increase on standing, often above 120 beats per mintes (bpm), accompanied by diffuse and non-specific symptoms such as deconditioning, cognitive impairment, fatigue, and gastrointestinal dysfunction^1^. POTS has been identified as one of the major cardiovascular phenotypes of post-acute COVID-19 syndrome (PACS), found in approximately 30% of highly symptomatic patients with PACS^2–4^. Since the pandemic there has been a rise in new POTS diagnoses, and patients with post-COVID POTS who prolong their sick-leave due to lingering symptoms, could burden the healthcare system^4^. Various POTS etiologies have been hypothesized such as abnormal hyperadrenergic activation due to catecholamine excess, compensatory tachycardia due to peripheral autonomic neuropathy, and chronic autoinflammatory or autoimmune processes^5^. Treatment of POTS relies on the correct diagnosis, which is at times problematic due to limited awareness of the condition. Treatment is usually directed towards symptom and sign alleviation through coping strategies, fluid intake, compression garments, and pharmacological agents targeting heart rate and blood pressure stabilization^6^. Gastrointestinal functional disorders are among the most prevalent POTS-related symptoms^7^, and their treatment is not well established due to unknown underlying mechanisms.

During the last years, there has been a growing interest for the interactions between gut microbiota and the function of the nervous system, the so-called microbiota-gut-brain axis. The gut microbiota has several functions in the gut, e.g., metabolism of chemicals, production of vitamin B and K, and production of short-chain fatty acids (SCFAs) from fermented carbohydrates^8^. The xenobiotic molecules produced by bacteria can affect immune cells, enteroendocrine cells, and neurons and glial cells in the ganglia of the enteric nervous system (ENS), cells which in turn release inflammatory molecules, peptides, and neurotransmitters^8–10^. Sensory afferent nerves from the ENS mediate information to the brain, with efferent autonomic nerves in the vagus nerve and sacral nerves mediating information from the brain back to the gut^8^. Circulating molecules may act directly on the central nervous system (CNS) as well^9^. The intestinal transit time affect the microbiota composition and thereby also the exposure of body tissue to the molecular products derived from the bacteria^9^. Therefore, all diseases affecting the gastrointestinal motility should affect the gut microbiota composition due to altered transit time. Only one study has examined the composition in gut microbiota in POTS compared with healthy controls, and that study could not find any associations between microbiota compositions and POTS^11^. However, clear differences between diabetes patients with and without autonomic neuropathy have been observed at all levels of the microbiota hierarchy^12^. Several studies have described how dysbiosis may be involved in the body`s adjustment after COVID-19, and perhaps even involved in long-term consequences of the disease (4).

In this study, we planned to explore the gut microbiota composition in relation to POTS and PACS to better understand the complexity of the diseases, and to try to identify mechanisms behind the gastrointestinal symptoms and fatigue.

## Material and methods

### Study design

Study participants with POTS were recruited at the Skåne University Hospital, Malmö, Sweden between October 2020 and January 2022. Invitations including study information were sent to patients with previously confirmed POTS at the Department of Cardiology at the same hospital, as described previously^13^. During the same time frame, healthy controls were recruited among hospital staff, their relatives, and students (*n* = 24). Study participants with PACS were recruited at Skåne University Hospital, Lund, Sweden between December 2021 and October 2022. Fifteen controls were recruited from a cohort of patients who had had COVID-19 but had no symptoms of PACS. All study participants were asked to complete a 4-day food diary at home. During the study visit, the participants were asked to complete a general study questionnaire and questionnaires regarding gastrointestinal symptoms, and a clinical examination was performed. Feces was collected at home in sterile tubes (Sarstedt, Nümbrecht, Germany), stored in the deep‐freezer until delivery to the hospital, then stored at − 80 °C until analyzed for gut microbiota composition at Clinical Microbiomics, Copenhagen, Denmark.

### Study populations

#### POTS patients

A POTS sub-study was established based on the Syncope Study of Unselected Population in Malmö (SYSTEMA) cohort at the Department of Cardiology between 2008 and 2021. Patients underwent cardiovascular autonomic tests with head-up tilt (HUT) testing with continuous hemodynamic monitoring, as well as other cardiac tests including ambulatory electrocardiogram (ECG) or 24-h ambulatory blood pressure monitoring, when appropriate. SYSTEMA has been previously described in detail^14^. The HUT testing protocol included supine rest for 10 min preceding table elevation to 60°–70° for 20 min for patients with suspected POTS and pronounced orthostatic intolerance^7^. POTS diagnosis was defined as symptoms of orthostatic intolerance lasting for ≥ 3 months associated with pathological HUT test showing heart rate (HR) increase > 30 bpm and absence of orthostatic hypotension^1^. From POTS substudy (*n* = 93), 82 patients were invited to be included in the present study since they were living within a reasonable proximity to Malmö, Sweden, were in the age between 18 and 70 years, and had the ability to fully understand the study information^13^. (Supplementary Fig. 1).

#### PACS-patients

Patients aged 18–70 years who had a confirmed infection with SARS-CoV-2 (positive PCR test, positive antigen test or positive serology without any other known cause), reported persisting symptoms ≥ 12 weeks after the infection^15^ and who had visited the PACS outpatient clinic at the Department of Infectious Diseases at Skåne University Hospital were eligible for recruitment. The symptoms were those commonly seen in PACS, including fatigue, neurologic and cognitive impairment, and chest symptoms^16^. Both patients who were admitted to hospital during the initial infection with SARS-CoV-2 and patients who had a milder infection which not required hospital admission were recruited in the study. The flow chart for inclusion is displayed in Supplementary Fig. 1.

#### Control subjects

Healthy control participants (*n* = 24) were first recruited among hospital staff and their relatives and medical students at Skåne University Hospital, Malmö, through personal invitation and advertisement. It is not known whether these 24 subjects have had COVID-19 or not. These controls were not allowed to have any current chronic or acute illness or significant gastrointestinal symptoms. Intake of multivitamins and hormonal contraceptive medicines was accepted, but otherwise, only temporary use of medications, such as seasonal allergy medicines, was allowed^13^. In addition, 15 extra controls were recruited by mail from the department Department of Infectious Diseases at Skåne University Hospital. These were subjects who had confirmed SARS-CoV-2 but who felt completely recovered and experienced no symptoms of PACS. Thus, a total of 39 controls with no diagnosis of POTS or PACS participated in the current study.

### Questionnaires

#### Study questionnaire

All study participants were asked to complete a questionnaire regarding sociodemographic factors, lifestyle habits, pregnancies and childbirth, previous and current illnesses, family history, and current pharmacological treatment.

#### Food diary questionnaire

The study participants were asked to keep a dietary record of all ingested food and drinks as well as associated gastrointestinal symptoms for 4 days. Each ingested item should be as thoroughly described as possible^17^. The food intake was grouped into categories of meat, fish, vegetables/legumes, fruits/berries, dairy products, cereal, candy, sugar-rich soda, sugar-free soda, juice, protein drinks, and energy drinks including fluid replacement by a senior physician (BO). The number of times they ingested any fast food or calorie-containing liquid was registered, as well as whether they had regular or un-regular food habits.

#### Gastrointestinal symptoms

The irritable bowel syndrome-severity scoring system (IBS-SSS) is a validated questionnaire for assessment of abdominal pain, abdominal distension, bowel habit satisfaction, and the impact of bowel habits on daily life using visual analog scales (VAS) between 0 and 100 mm, where 100 mm means very severe symptoms. Abdominal pain frequency in the last 10 days is registered. The combined maximum total score is 500. Scores of 75–174 indicate mild, 175–299 moderate, and ≥ 300 severe disease. Also, the most common extraintestinal symptoms in IBS is assessed on VAS scales^18^.

The visual analog scale for irritable bowel syndrome (VAS-IBS) is a validated questionnaire regarding abdominal pain, diarrhea, constipation, bloating and flatulence, vomiting and nausea, the intestinal symptoms’ influence on daily life, and psychological well-being during the last 2 weeks. The symptoms are assessed on VAS scales between 0 and 100 mm, where 100 mm means very severe symptoms. The scales are inverted from the original version^19^.

### Microbiota analysis

#### DNA extraction

DNA was extracted from ~ 0.1 g aliquots of the fecal samples using the NucleoSpin 96 Soil (Macherey–Nagel, Hoerdt, Germany) kit. Bead beating was done horizontally on a Vortex-Genie 2 at 2700 rpm for 2 × 5 min. The samples were almost intact after only 5 min (standard) bead beating; the stool consistency was dry and hard. Double bead beating improved the DNA concentrations of the samples. Two samples did not have sufficient material for re-extraction and were therefore only subjected to 5 min bead beating. A minimum of one negative control was included per batch of samples from the DNA extraction step and throughout the laboratory process (including sequencing). A ZymoBIOMICS Microbial Community Standard (Zymo Research, Freiburg im Breisgau, Germany) was included in the analysis as a positive (mock) control.

#### DNA sequencing

Before library preparation, the DNA was quantified by Tecan Infinite F Nano + Plate Reader (Tecan, Maennedorf, Switzerland) using Quant-iT dsDNA BR Assay Kit (Thermo Fisher Scientific, Waltham, MA, USA). The enzymatic fragmentation of DNA and library construction was conducted by Tecan DreamPrep NGS using Celero EZ DNA -seq Core Module Kit (Tecan). The fragmented DNA was amplified using PCR. Short and large DNA fragments were removed using double-sided magnetic bead size selection (AMPure XP, Beckman Coulter, Indianapolis, Indiana, USA). Adapter sequences from Celero 96-Plex Adaptor Plate (Tecan) were added to each sample during library construction. The final concentration for each library was quantified by Tecan Infinite F Nano + Plate Reader using NuQuant NGS Library Quantification Module and Qubit (Tecan). The final fragment distribution was evaluated using a Fragment Analyzer 5200 (Agilent, Santa Clara, CA, USA). Qubit and TapeStation were used to determine the concentration of 10% of the final library before sequencing. The library was sequenced using 2 × 150 bp paired-end sequencing on an Illumina platform (Rome, Italy).

#### Gene catalog and metagenomic species definitions

The Clinical Microbiomics Human Gut HG04 gene catalog (14 355 839 genes) was used as a reference gene catalog, which was created based on 12 170 non-public deep-sequenced human gut specimens (including 481 from infants), 9428 publicly available metagenomes compiled from 43 countries^20^, and 3567 publicly available genome assemblies from isolated microbial strains. For taxonomic abundance profiling, the Clinical Microbiomics HGMGS version HG4.D.2 set of 2 095 metagenomic species (MGS) was used, each represented by a set of genes with highly coherent abundance profiles and base compositions in the 12 170 metagenomes. The metagenomic species concept is described in Nielsen et al. 2014^21^. To taxonomically annotate an MGS, its genes were blasted against NCBI RefSeq archaea, bacteria, fungal, protozoa, and viral genomes (2022–01-19) and nt (2021–08-03) databases with rank-specific annotation criteria. Definitions used: PID = (95, 95, 85, 75, 65, 55, 50, 45); M = (75, 75, 60, 50, 40, 30, 25, 20); and D = (10, 10, 10, 20, 20, 20, 20, 15) for subspecies, species, genus, family, order, class, phylum, and superkingdom (domain), respectively. Specifically, a taxon was assigned to an MGS if at least M% of its genes were mapped to the taxon and no more than D% of its genes were mapped to a different taxon. Blast hits were only considered if there was an alignment length ≥ 100 bp, ≥ 50% query coverage, and % identity ≥ PID. Finally, each MGS was processed with CheckM^22^ and the annotation was updated with the CheckM result if this resulted in a lower taxonomic rank.

#### Sequencing data preprocessing

Raw FASTQ files were filtered to remove host contamination by discarding read pairs in which either of the two paired reads mapped to the human reference genome GRCh38 with Bowtie2 v. 2.4.2^23^. Reads were then trimmed to remove adapters and bases with a Phred score below 30 using AdapterRemoval v. 2.3.1^24^. Read pairs in which both reads passed filtering with a length of at least 100 bp were retained; these were classified as high-quality non-host (HQNH) reads.

#### Mapping reads to the gene catalog

HQNH reads were mapped to the gene catalog using BWA mem v. 0.7.17^25^. An individual read was considered uniquely mapped to a gene if the mapping quality (MAPQ) was ≥ 20 and the read aligned with ≥ 95% identity over ≥ 100 bp. However, if > 10 bases of the read did not align to the gene or extend beyond the gene, the read was considered unmapped. Reads meeting the alignment length and identity criteria but not the MAPQ threshold were considered multi-mapped. Each read pair was counted as either (1) uniquely mapped to a specific gene, if one or both individual reads were uniquely mapped to a gene, or (2) multi-mapped, if neither read was uniquely mapped, and at least one was multi-mapped, or (3) unmapped, if both individual reads were unmapped. If the two reads were each uniquely mapped to a different gene, the gene mapped by read 1 was counted but not the gene mapped by read 2. A gene count table was created with the number of uniquely mapped read pairs for each gene.

#### MGS relative abundance calculation

For each MGS, a signature gene set was defined as the 100 genes optimized for accurate abundance profiling of the MGS. An MGS count table was created by counting the number of reads uniquely mapped to the MGS signature genes per sample (lundys_r1v4_all-tables.xlsx, sheet “MGS-counts”). An MGS was considered detected if reads from a sample uniquely mapped to at least three of its signature genes; measurements that did not satisfy this criterion were set to zero. Based on internal benchmarks, this threshold results in 99.6% specificity. The MGS count table was normalized according to effective gene length and then normalized sample-wise to sum to 100%, resulting in relative abundance estimates for each MGS (lundys_r1v4_all-tables.xlsx, sheet “MGS-relative-abundance”). Down-sampled (rarefied) MGS abundance profiles were calculated by random sampling, without replacement, of a fixed number of signature gene counts per sample, and then following the procedure described above (lundys_r1v4_all-tables.xlsx, sheets “MGS-downsized-counts” and “MGS-downsized-relative-abundance”). In this study, 70 855 signature gene counts were sampled.

The metagenomic analysis was performed using the MGS concept^21^ and the Clinical Microbiomics human gut MGS database.

#### Functional annotation and profiling

EggNOG-mapper v. 2.1.7, Diamond mode^26^ was used to map each gene in the gene catalog to the EggNOG orthologous groups database v. 5.0^27^, resulting in Kyoto Encyclopedia of Genes and Genomes (KEGG) orthology (KO) database annotations for 39% of genes. Functional potential profiles based on KOs were calculated as the proportion of all mapped reads that mapped to a given KO. KEGG modules v. 78.2^28^ are defined as a set of KOs that enable a specific function or pathway. For each KEGG module, its corresponding FSG was defined as the set of MGSs that include at least 2/3 of the genes that encode the proteins/enzymes that are needed to complete the functionality of the module. If a module has alternative reaction paths, only one of these is required to be 2/3 complete. For modules with three or fewer steps, all steps are required to be comprised in the MGS. The Gut Metabolic Modules (GMMs) are a set of 103 conserved metabolic pathways, each defined as a series of enzymatic steps represented by KEGG Orthology (KO) identifiers^29^. An MGS was considered to contain a given module if the MGS includes genes annotated to at least 2/3 of the KOs needed to complete the functionality of the module. If a module has alternative reaction paths, only one of these is required to be 2/3 complete. For modules with three or fewer steps, all steps are required to be comprised in the MGS.

#### Diversity estimates

Alpha and beta diversity estimates were calculated from rarefied abundance matrices, created by random sampling of reads without replacement. Within each data type (e.g., gene, MGS), all samples were represented by the same number of informative sequencing reads: rarefaction of MGS abundance was performed by sampling only from reads mapping to MGS signature genes. However, rarefaction of gene abundance was performed by sampling reads mapped to the entire gene catalog. Alpha diversity was calculated as the number of entities detected (richness), or as the Shannon index based on natural logarithm. Beta diversity was calculated as the Bray–Curtis dissimilarity, which accounts for differences in relative abundances of MGS among samples. Bray–Curtis dissimilarity can range between 0 and 1, where 0 means that the two samples have identical compositions (they share all species at the same relative abundance), and 1 means that the two samples are completely different (they do not share any species). For visual interpretation, the samples were projected onto the first two dimensions of a principal component analysis (PCoA) of the Bray–Curtis dissimilarities with the larger points representing centroids of a group of samples.

#### Statistical analyses

Differences between patients and controls regarding basal characteristics were calculated in SPSS, version 28, by Mann–Whitney *U* test or Fisher´s exact test. *P* < 0.05 was considered statistically significant.

The analysis of potential confounders was performed by initially testing the association between the given phenotypes and the beta diversity measure (Bray–Curtis dissimilarity) between samples. The association of beta-diversity with binary phenotypes was tested with permutational multivariate analysis of variance (PERMANOVA) using the adonis2 function from the vegan *R* package with 1000 permutations. The association of beta-diversity with continuous phenotypes was tested with distance-based redundancy analysis (db-RDA), using the anova.cca function from the vegan *R* package with 1000 permutations.

Phenotypes that were significantly associated to microbiome composition (beta diversity) were further tested for association with the diagnosis variables with a Mann–Whitney *U* test for the continuous phenotypes and a Chi-squared test for the binary phenotypes. The association was considered significant with a *P*-value lower than 0.05. Phenotypes associated with both the beta diversity and a diagnosis variable, were regarded as potential confounders, and adjusted for in the subsequent statistical testing.

For each taxonomic category and both group variables, a linear regression model was constructed with the logarithm of the relative abundance of the taxonomic category as the dependent variable, and the group variable, as well as the potential confounding variables, as the independent covariates. The MGS-level abundances were aggregated (summed).

$${\text{Log }}\left( {\text{Relative abundance of taxon}} \right) \, = \alpha + \beta_{{1}} {\text{POTS }} + \Sigma \beta_{i} {\text{Confounder}}_{i} +{\varepsilon }$$

$${\text{Log }}\left( {\text{Relative abundance of taxon}} \right) \, = \alpha + \beta_{{1}} {\text{PACS}} + \Sigma \beta_{i} {\text{Confounder}}_{i} +{\varepsilon }$$

The coefficient of the regression model, corresponding to the group variable as well as the coefficient’s standard deviation and *p*-value are reported. Only those taxa that were prevalent in at least 10% of the samples were considered. The correlation analysis was performed with Kendall’s method and with prevalence threshold of 10%.

When performing statistical testing on multiple hypotheses, the Benjamini–Hochberg (BH) method to control the false discovery rate (FDR) was used at a level of 10%. Thus, of all the “statistically significant” associations reported in this context, no more than 10% of these were expected to be false associations (arising due to chance). Thus, clinical variables were considered significantly associated with the microbiome variables if FDR was below 0.1. FDR control was applied to each tested contrast individually, i.e., it accounts for multiple species being tested but does not account for multiple contrasts. “Nominal” *P* value indicates that the *P* value was not subjected to FDR control nor otherwise adjusted for multiple hypotheses.

### Ethics approval

The present study was performed in accordance with the Declaration of Helsinki and approved by The Swedish Ethical Review Authority, Dnr 2020–02,432 and 2021–00,049 for POTS and 2021–03,905 for PACS.

### Patient consent

All study participants provided informed written consent prior inclusion.

## Results

### Basal characteristics

Forty-three patients with a diagnosis of POTS were initially included in the investigation to assess gastrointestinal symptoms. Twenty-seven of these patients, two of whom had received the POTS diagnosis after their COVID-19 infection, delivered fecal samples in which DNA could be retrieved and were thus included in the current study. Fifty-two patients with PACS were included in a study to assess long-term consequences of COVID-19, but three patients were excluded since they didn’t meet the inclusion criteria. Of these, 32 delivered fecal samples in which DNA could be analyzed and were included in the current study. Five of these patients fulfilled the diagnostic criteria of POTS^1^. All patients with POTS, independent of genesis, were assembled into the “POTS” cohort (*n* = 32) and all patients with PACS were assembled into the “PACS” cohort (*n* = 34), for comparison with controls with neither POTS nor PACS (*n* = 39) (Supplementary Fig. 1). The most frequent comorbidities and drug treatment is reported in Supplementary Table 1. One POTS patient registered concomitant diabetes. Six patients with PACS ingested probiotics regularly.

There were no differences in sex or smoking habits between patients and controls. The dietary intake of meat, fish, vegetables, fruits, dairy products, sugar-free soda, and fast food were equal between groups (data not shown), but the intake of candy and cereal was higher in controls than in patients, whereas the sugar-rich soda intake was higher in POTS patients than in controls. Both POTS and PACS patients had more gastrointestinal symptoms and fatigue than controls. The most common gastrointestinal symptom was bloating and flatulence (Table 1). All data is shown in supplementary file lundys_r1v3_metadata.xlsx.

**Table 1:** Basal characteristics.

	POTS (*n* = 32)	PACS (*n* = 34)	Controls (*n* = 39)	*P*-value POTS	*P*-value PACS
Female sex (*n*, %)	30 (93.9)	26 (76.5)	30 (76.9)	0.096	1.00
Smokers (*n*, %) Missing value		3	2	0.221	0.262
Regularly	2 (6.3)	0 (0)	0 (0)		
Sometimes	0 (0)	0 (0)	3 (8.1)		
Former smoker	6 (18.8)	9 (26.5)	7 (17.9)		
Never smoked	24 (75.0)	22 (64.7)	27 (73.0)		
Sugar-rich soda (intakes/4 days) missing value	0 (0–1) 3	0 (0–0)	0 (0–0) 5	0.013	0.787
Candies (intakes/4 days) missing value	2 (1–4) 3	2 (0–4)	4 (2–5) 5	0.022	0.001
Cereals (intakes/4 days) missing value	6 (4–8) 3	6 (2–8)	8 (6–11)	0.008	0.005
IBS-SSS missing value	189 (46–278) 3	74 (30–178) 3	7 (0–58) 3	< 0.001	< 0.001
Abdominal pain (mm) missing value	30 (0–62)	0 (0–32) 1	0 (0–0) 2	< 0.001	0.010
Diarrhea (mm) missing value	28 (0–69)	0 (0–38) 1	0 (0–0) 2	< 0.001	0.014
Constipation (mm) missing value	26 (0–72)	16 (0–55) 2	0 (0–16) 2	< 0.001	0.004
Bloating and flatulence (mm) missing value	64 (18–89)	19 (2–53) 1	0 (0–8) 3	< 0.001	< 0.001
Vomiting and nausea (mm) missing value	36 (22–68)	9 (0–30) 1	0 (0–3) 3	< 0.001	0.002
Psychological well-being (mm) missing value	50 (30–60)	46 (22–60) 1	0 (0–17) 4	< 0.001	< 0.001
Intestinal symptom´s influence on daily life (mm) missing value	51 (18–76) 1	18 (0–46) 2	0 (0–2) 3	< 0.001	< 0.001
Fatigue (mm) missing value	94 (86–100)	82 (64–98) 1	12 (0–30) 3	< 0.001	< 0.001

IBS = irritable bowel syndrome, PACS = post-acute COVID-19 syndrome, POTS = postural orthostatic tachycardia syndrome. Total IBS-Severity Scoring System (SSS) is calculated after Francis et al. (15). Specific gastrointestinal symptoms and fatigue are assessed by the visual analog scale for IBS (VAS-IBS) (16). Symptoms are assessed on VAS scales 0–100 mm, where 0 means no symptoms and 100 maximal symptoms (15, 16). Comparisons were made between controls and patients with POTS or PACS by Mann–Whitney *U* test or Fisher´s exact test. Some patients are included in both cohorts. Values are given as number and percentages or median and interquartile values. *P* < 0.05 was considered statistically significant.

### Sequencing results and taxonomic overview

The sequencing data was good from all samples, with an average of 28.3 M read pairs per sample and a minimum of 1.8 M read pairs (Supplementary Fig. 2). Proportion of read pairs can be seen in Supplementary Fig. 3. On average 22.6 M read pairs per sample could be mapped to the gene catalog, representing on average 81.4% of the HQNH reads (min = 76.3%, Supplementary Table 2). Sequencing read quality control and gene catalog mapping statistics for each sample can be found in the supplementary file lundys_r1v4_QC_summary.xlsx.

**Figure 1:**
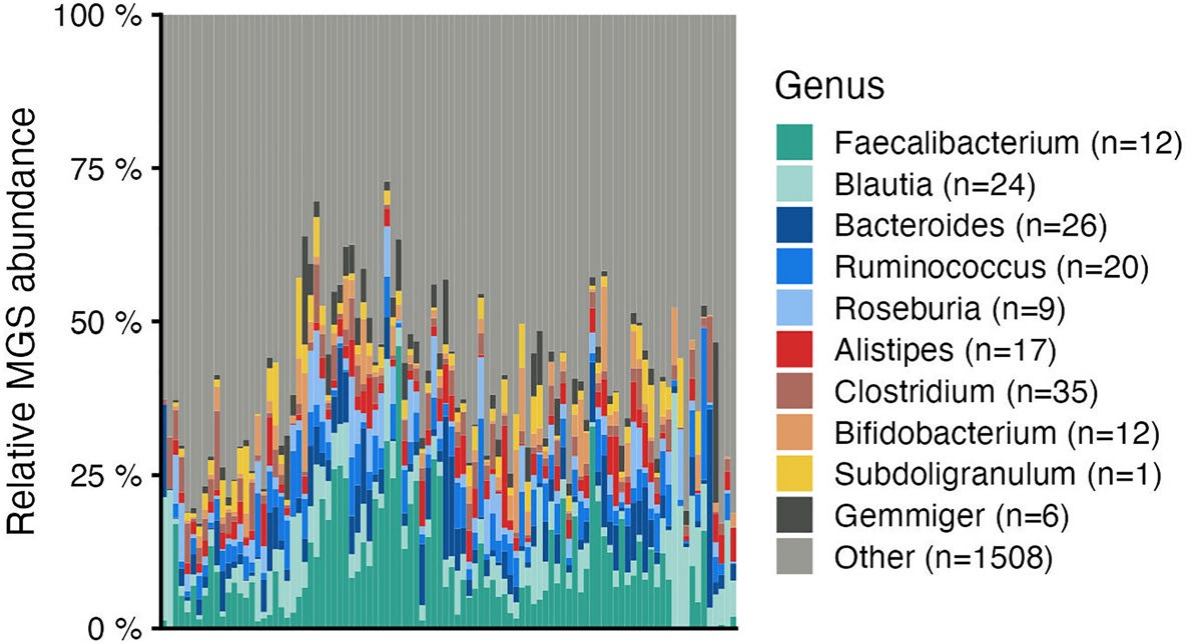
Taxonomic overview at genus level per sample. Bar plots display the relative abundance of the top 10 taxa with highest average abundance across all samples. Light grey (Other) indicates the total relative abundance of metagenomic species (MGSs) that are not in the top 10 most abundant taxa.

**Figure 2:**
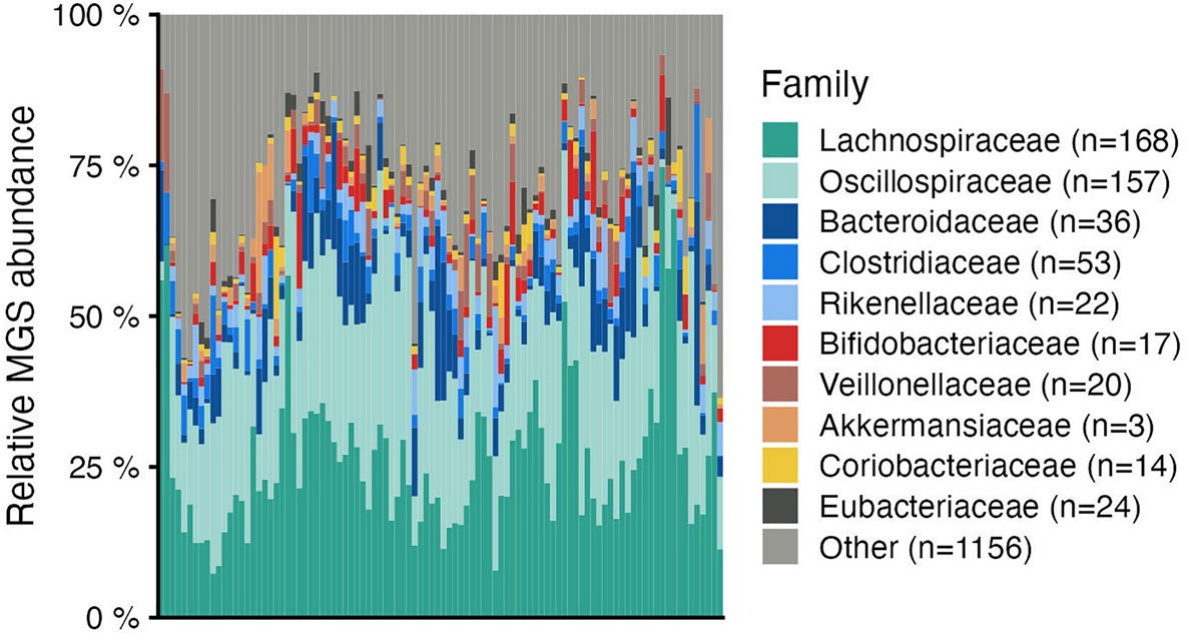
Taxonomic overview at family level per sample. Bar plots display the relative abundance of the top 10 taxa with highest average abundance across all samples. Light grey (Other) indicates the total relative abundance of metagenomic species (MGSs) that are not in the top 10 most abundant taxa.

The relative abundances for all MGSs across all samples were determined and Gene-based KO profiles, MGS-based KEGG-module profiles and MGS-based GMM profiles were generated for each sample using KO database (supplementary file lundys_r1v4_all-tables.xlsx). Taxonomic profiles aggregated at genus level per sample are shown in Fig. 1 and taxonomic profiles aggregated at family level are shown in Fig. 2.

### Confounder analysis

**Figure 3:**
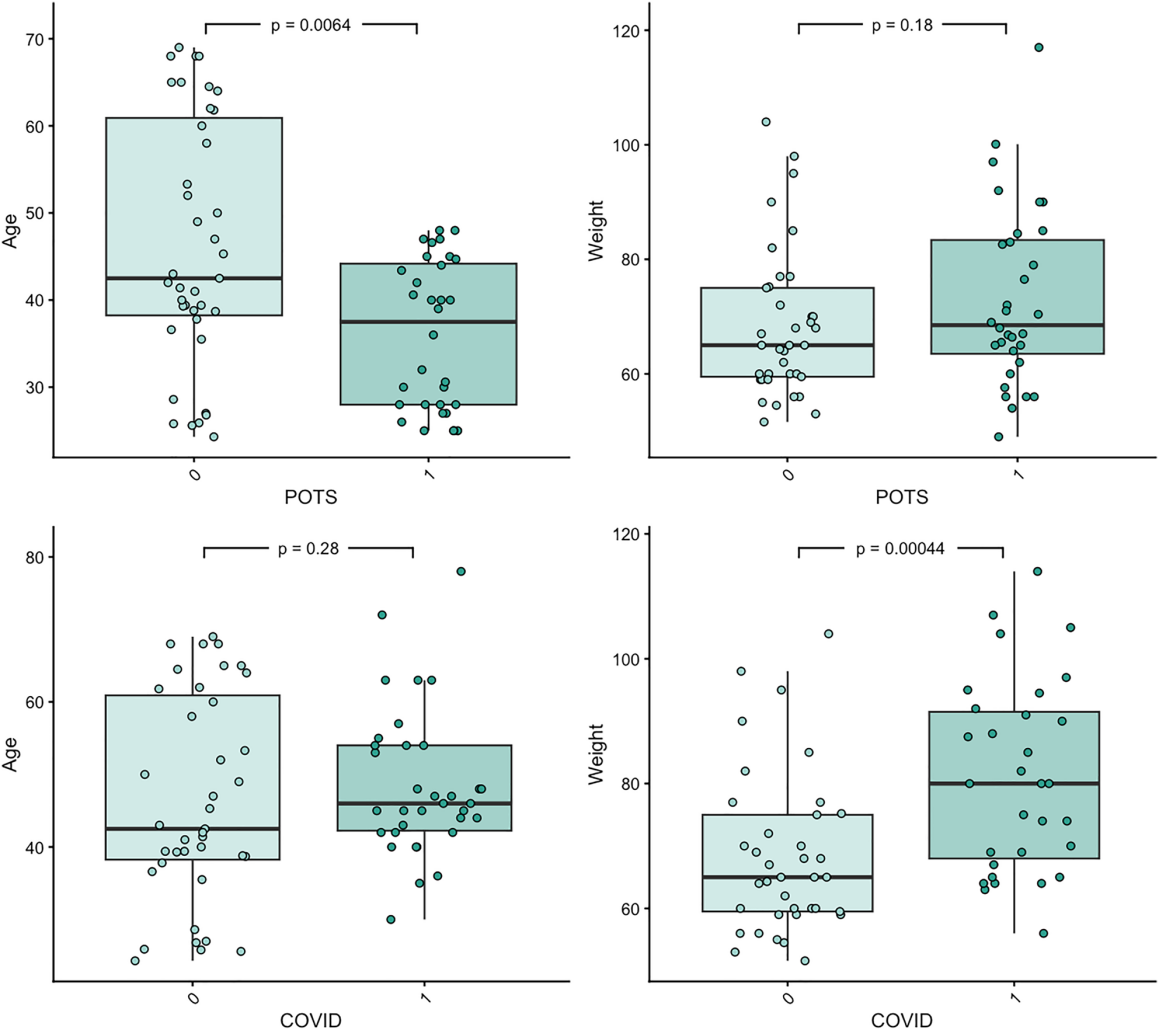
Boxplots showing the difference in age and weight between patients with Postural Orthostatic Tachycardia Syndrome (POTS) and controls and between patients with post-acute COVID-19 syndrome (PACS) and controls. Mann–Whitney *U* test. *P* < 0.05 was considered statistically significant.

The phenotypes age, sex, weight, smoking habit, and consumption of sugar-rich soda were investigated for their potential confounding effects (supplementary file lundys_r1v4_confounder_analysis_results.xlsx). Age and weight were found to be significantly associated to the Bray–Curtis dissimilarity (age: FDR = 0.062, weight: FDR = 0.02). Age and weight were further tested for their association with the group variables POTS and PACS with a Mann–Whitney-*U* test. Age was significantly associated with POTS (*P* = 0.006) and weight was significantly associated with PACS (*P* = 0.00044) (Fig. 3), and therefore adjusted for in subsequent statistical models.

### Changes in microbiome composition

**Figure 4:**
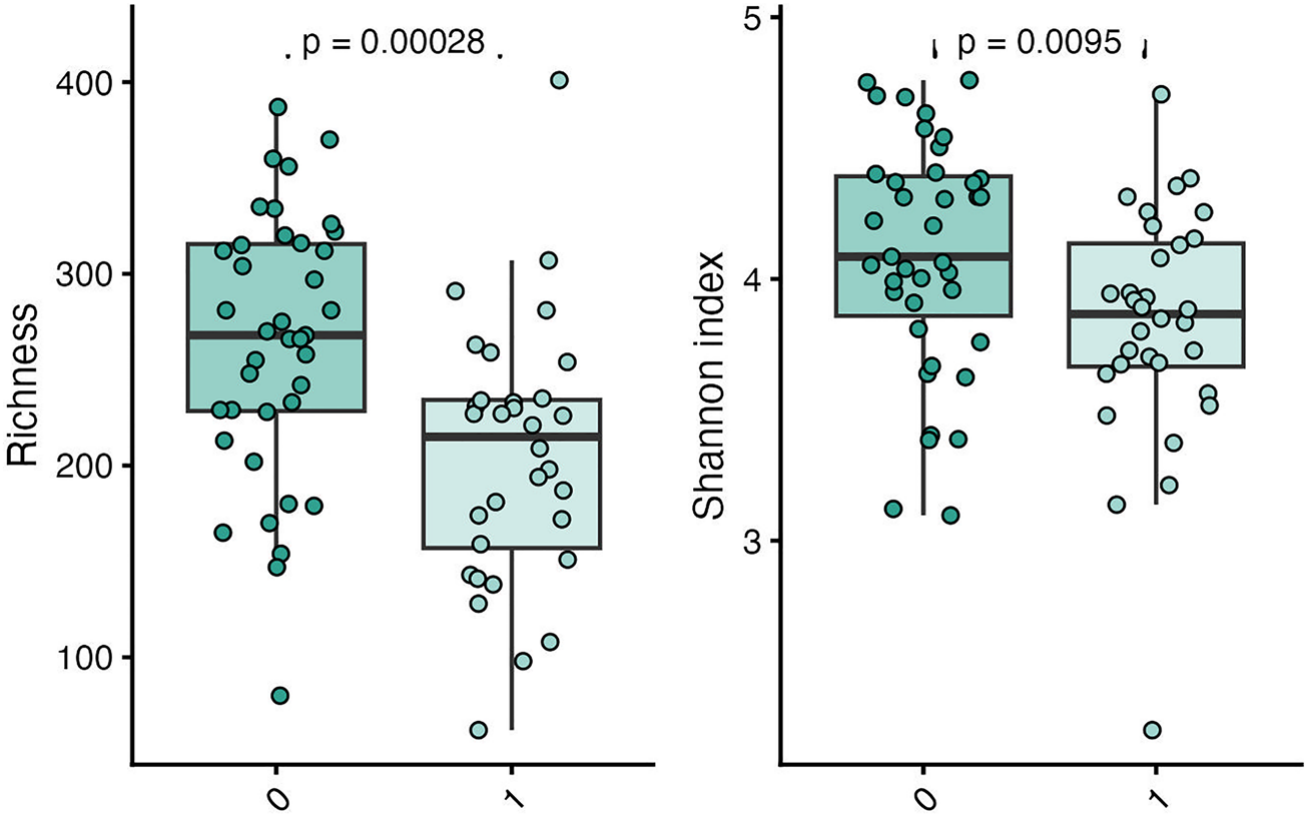
Boxplots showing differences in MGS richness and Shannon diversity grouped by POTS or not. The groups were compared pairwise by Mann–Whitney *U* test. Both alpha diversity measures were significantly lower in the POTS group than in the control group (Richness: *P* = 0.00028, Shannon index: *P* = 0.0095).

**Figure 5:**
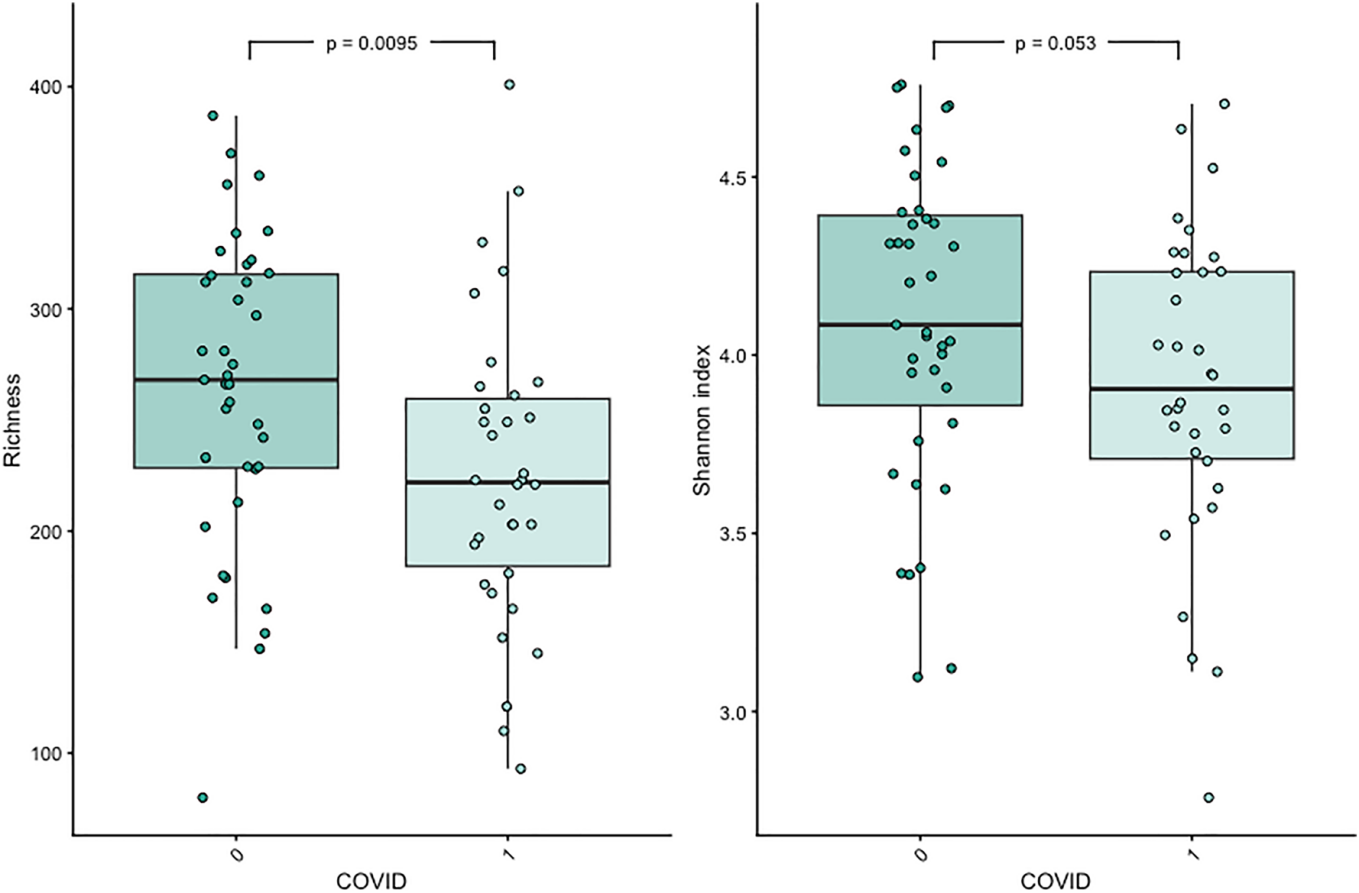
Boxplots showing differences in MGS richness and Shannon diversity grouped by post-acute COVID-19 syndrome (PACS) or not. The groups were compared pairwise by Mann–Whitney *U* test. Richness was significantly lower in the PACS group compared to controls (*P* = 0.0095).

Alpha diversity calculated as richness and Shannon index were significantly lower in individuals with POTS compared to controls (Fig. 4), whereas only richness was significantly lower in individuals with PACS compared to controls (Fig. 5). The alpha diversity measures for each sample are shown in supplementary files lundys_r1v4_alpha-diversity.xlsx.

**Figure 6:**
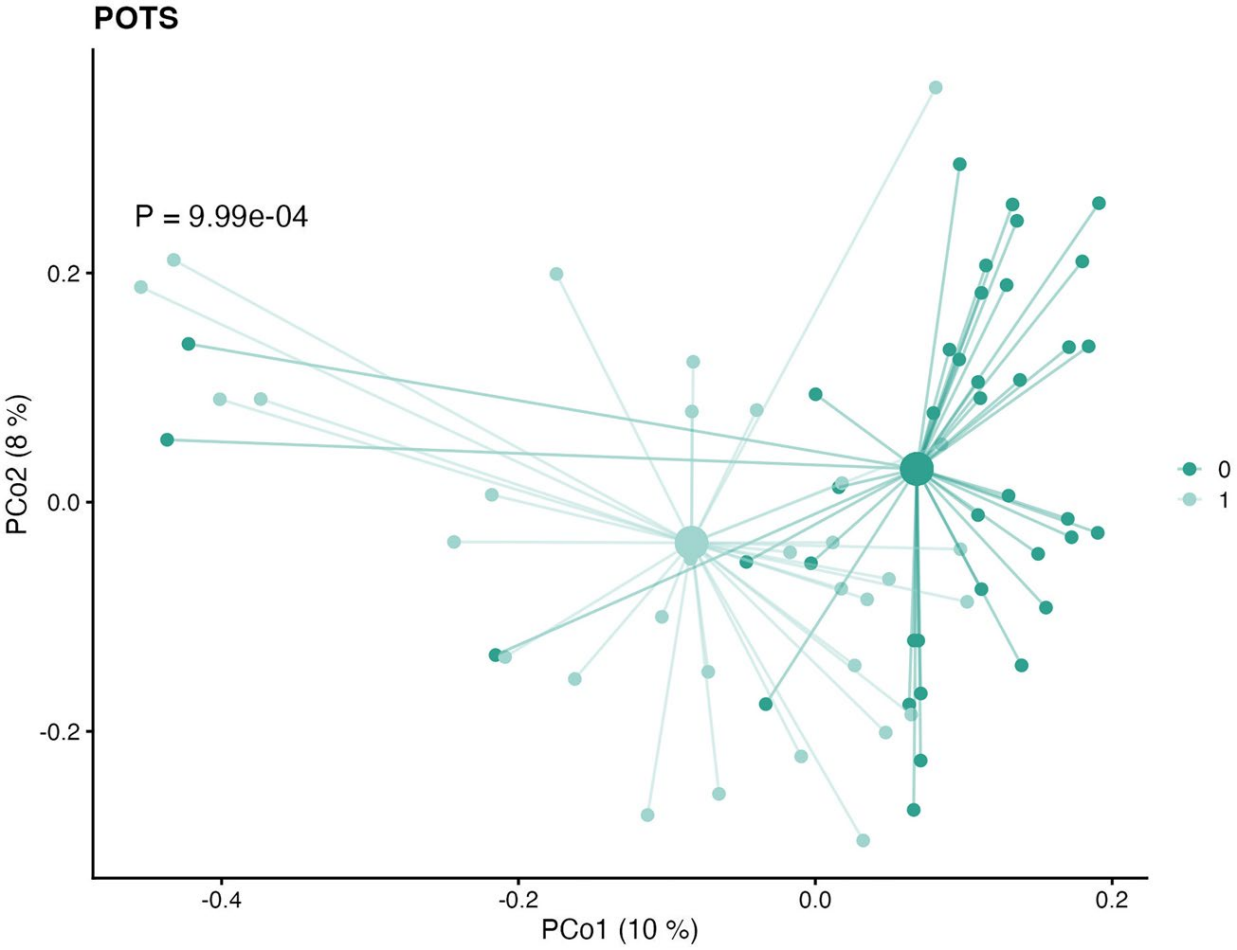
Bray–Curtis dissimilarities among samples, calculated based on the metagenomic species (MGS) abundances. The mean (centroid) of samples in each group (Postural Orthostatic Tachycardia Syndrome (POTS) or controls) is indicated with a larger shape. Each sample is connected to its centroid by a thin line. The *x*-and *y*-axis labels indicate the microbial variance explained by the first two principal coordinates. The microbiome composition significantly differs between groups (*P* = 9.99e-04).

**Figure 7:**
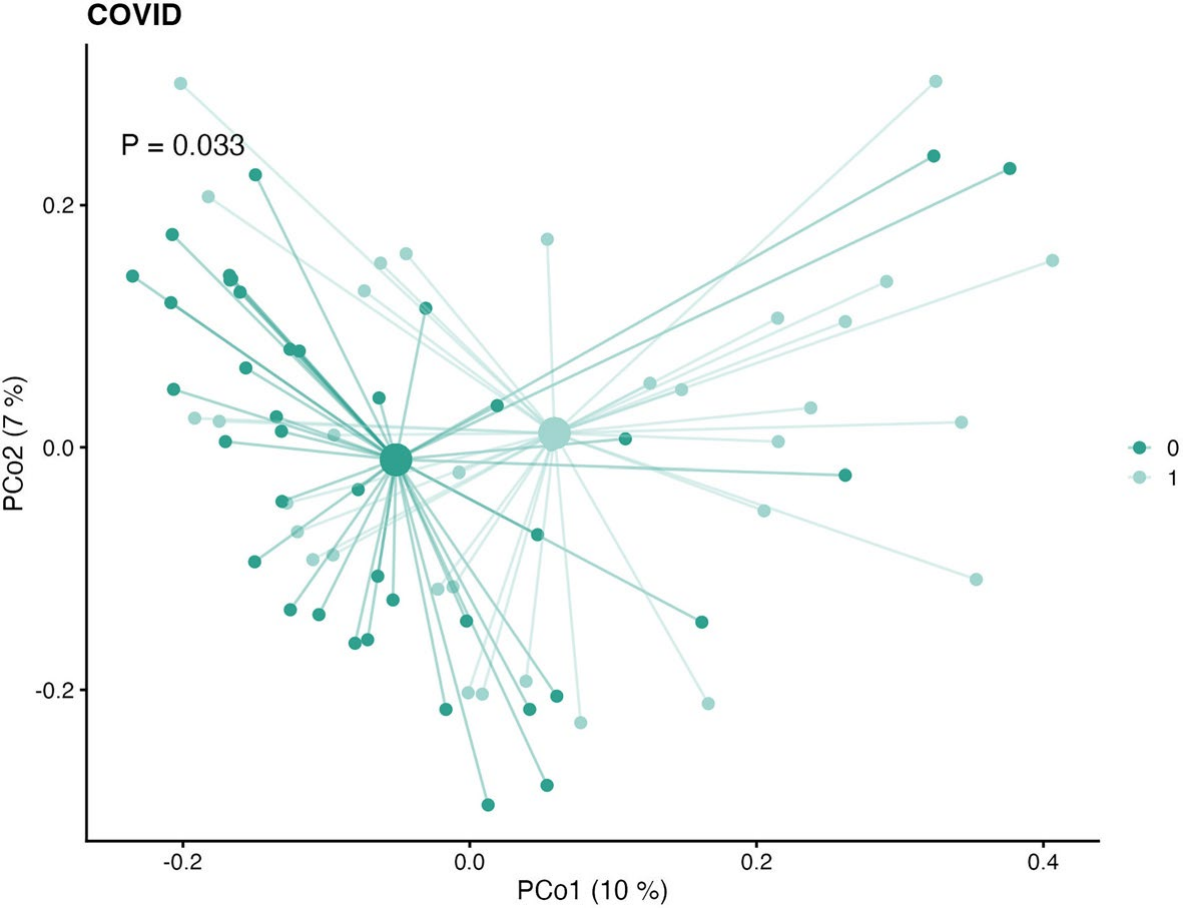
Bray–Curtis dissimilarities among samples, calculated based on the metagenomic species (MGS) abundances. The mean (centroid) of samples in each group (post-acute COVID-19 syndrome (PACS) or controls) is indicated with a larger shape. Each sample is connected to its centroid by a thin line. The *x*-and *y*-axis labels indicate the microbial variance explained by the first two principal coordinates. The microbiome composition significantly differs between groups (*P* = 0.033).

The overall changes in microbiome community composition were significantly different between individuals with POTS and controls (*P* = 9.99e-04) (Fig. 6). Similarly, the microbiome composition also significantly differed between individuals in the PACS group compared to controls (*P* = 0.033) (Fig. 7). The sample-wise beta diversity tables are shown in supplementary files lundys_r1v4_beta-diversity.xlsx.

In the POTS group, the abundance of several bacteria at the MGS, species, and genus level, and a few bacteria at the family and order level, differed significantly compared with controls (Table 2 and supplementary file lundys_r1v4_POTS_regression_test_results.xlsx). The largest effect sizes for POTS at the MGS level is shown in Table 3, with lowest FDR for *Eubacteriales.sp*, *Faecalibacterium prausnitzii,* and *Faecalibacterium sp*.

**Table 2:** Overview over regression analysis from POTS group.

Category	Number of taxa	Number of significant taxa
MGS	689	112
Subspecies	17	0
Species	287	50
Genus	138	25
Family	43	5
Order	27	3
Class	18	0
Phylum	12	0
Superkingdom	3	0

POTS = Postural Orthostatic Tachycardia Syndrome A taxon was counted as significant if the false discovery rate (FDR) adjusted *p*-value for the coefficient for group in the corresponding model was below 0.1. The FDR correction was done per taxonomic category.

**Table 3:** MGS taxa associated with POTS.

Taxonomy	Regression coefficient	*P*-value	FDR	Median abundance (%)		Prevalence	
				Controls *N* = 39	POTS *N* = 32	Controls *N* = 39	POTS *N* = 32
HG4D.0062 eubacteriales sp.	− 3.300	3.66e-05	0.008	0.455	0.00e + 00	34	15
HG4D.0029 faecalibacterium prausnitzii	− 2.860	0.002	0.039	1.104	0.205	37	22
HG4D.0042 faecalibacterium sp.	− 2.539	1.74e-04	0.014	1.361	0.229	37	18
HG4D.0019 collinsella sp.	− 2.019	0.003	0.044	0.806	0.372	37	23
HG4D.0061-ruminococcus sp.	− 2.014	0.013	0.087	0.108	0.041	35	16
HG4D.0068 Eubacterium ramulus	− 1.912	0.006	0.062	0.080	0.033	36	19
HG4D.0063 blautia sp. SG-772	− 1.562	0.005	0.055	0.195	0.109	36	20
HG4D.0043 fusicatenibacter sp. CLA-AA-H277	− 1.373	0.002	0.040	0.063	0.031	37	22
HG4D.0057 oscillospiraceae sp.	− 1.366	0.007	0.065	0.143	0.090	35	21
HG4D.0020 –ruthenibacterium lactatiformans	0.817	0.006	0.062	0.077	0.204	39	32

FDR = false discovery rate, MGS = Metagenomic Species, POTS = Postural Orthostatic Tachycardia Syndrome. The median relative abundance (in %) of each taxon in each group is shown. Prevalence is the number of samples with a nonzero abundance for the given taxon. The effect size is represented as the coefficient for the group variable POTS in the linear regression model where the relative abundance is log transformed. Positive values imply the taxon is more abundant in the patients group compared to the controls. The FDR adjusted *P*-value, adjusted for all tests, were performed within each taxonomic category and considered statistically significant if < 0.1. Redundant taxa have been omitted from the table.

At the species level, the largest positive associations with POTS were found for *Enterocloster bolteae* (FDR = 0.008), *Clostridium innocuum, Eggerthella Lenta* and *Gordonibacter urolithinfaciens* (FDR = 0.01), *Massilimicrobiota timonensis* (FDR = 0.017), *Enterocloster aldenensis* (FDR = 0.024), *Gordonibacter pamelaeae* and *Hungatella hathewayi* (FDR = 0.017), *Anaerotruncus colihominis* and *Sellimonas intestinalis* (FDR = 0.024), *Lachnoclostridium sp. An138* (FDR = 0.037), *Enterocloster citroniae* and *Flavonifractor plautii* (FDR = 0.039), *Clostridium symbosium* (FDR = 0.048), *Eisenbergiella massiliensis* (FDR = 0.05), *Ruminococcus gnavus, Blautia hydrogenotrophica,* and *Blautia producta* (FDR = 0.058), *Lactiplantibacillus plantarum* (FDR = 0.076), *Clostridium scindens*, *Enterocloster clostridioformis, Dielma fastidiosa,* and *Merdimonas faecis* (FDR = 0.078), and *Erysipelatoclostridium ramosum* (FDR = 0.09). The largest negative effect sizes were observed for *Haemophilus parainfluenzae* (FDR = 0.025), *Alistipes sp. AF17-16, Ellagibacter isourolithinifaciens, Faecalibacterium sp. CLA-AA-H233, Fusicatenibacter sp. CLA-AA-H277,* and *Ruminococcus sp. AF21-42* (FDR = 0.037), *Anaerotignum faecicola* and *Faecalibacterium sp. OF04-11AC* (FDR = 0.038), *Allistipes senegalensis*, and *Ruminococcus sp. AF46-10NS* (FDR = 0.039), *Clostridium sp. AM49-4BH* (FDR = 0.048) *Blautia sp. SG-772* (FDR = 0.05), *Phascolarctobacterium succinatutens* (FDR = 0.053), *Blautia sp. BIOML-A1, Ruthenibacterium lactatiformans,* and *Senegalimassilia anaerobia* (FDR = 0.058), *Faecalibacterium prausnitzii*, and *Slackia isoflavoniconvertens* (FDR = 0.076), *Ruminococcus sp. AF41-9* (FDR = 0.078), *Eubacterium ventriosum, Coprococcus eutactus, Roseburia sp. OM04-15AA,* and *Ruminococcus sp. AF17-22AC* (FDR = 0.081), *Bacteroides faecis* (FDR = 0.09), *Allistipes communis* and *Prevotella rara* (FDR = 0.10).

**Figure 8:**
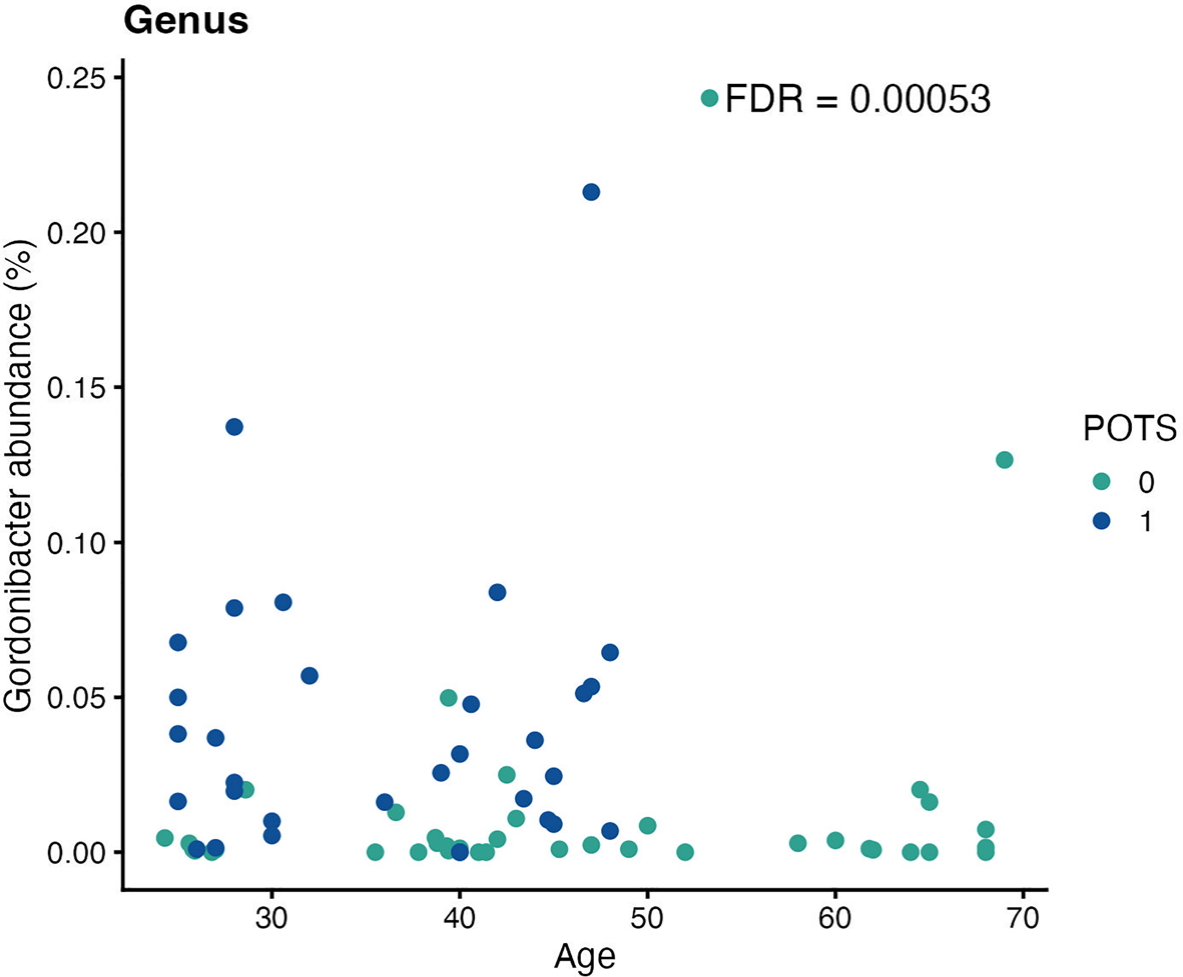
Scatter plot showing the confounding effect (age) on the relative genus abundance. Colours indicate the different groups (0 = controls, 1 = Postural Orthostatic Tachycardia Syndrome (POTS)). Even though individuals with POTS are generally younger, there was still a significant difference in *Gordonibacter* abundance between groups (False discovery rate (FDR) = 0.001).

At the genus level, the largest positive associations with POTS were found for *Enterocloster* and *Gordonibacter* (FDR = 0.001) (Fig. 8), *Erysipelatoclostridium* (FDR = 0.002), *Anaerotruncus* (FDR = 0.004), *Eggerthella* (FDR = 0.005), *Hungatella* and *Massilimicrobiota* (FDR = 0.007), *Sellimonas* (FDR = 0.011), *Flavonifractor* (FDR = 0.017), *Faecalicatena* (FDR = 0.045), *Ruthenibacterium* (FDR = 0.049), *Bacteroides* (FDR = 0.057), *Holdemania* (FDR = 0.059), *Dielma* and *Lactiplantibacillus* (FDR = 0.065), *Merdimonas* (FDR = 0.068), and *Eisenbergiella* (FDR = 0.085). Negative associations were observed for *Phascolarctobacterium* (FDR = 0.01), *Haemophilus* (FDR = 0.011), *Prevotella* (FDR = 0.017), *Elligabacter* (FDR = 0.023), *Collinsella* (FDR = 0.032), *Holdemanella* (FDR = 0.045), *Senegalimassilia* (FDR = 0.052), and *Anaerotignum* (FDR = 0.08).

The largest effect size at family levels between POTS and control were observed for *Prevotellaceae, Acidaminococcaceae,* and *Atopobiaceae* with negative associations (Table 4). *Pasteurellales, Coriobacteriales,* and *Fusobacteriales* were the only bacteria at the order level which showed associations (negative) with POTS (FDR = 0.019, 0.056, and 0.093, respectively).

**Table 4:** Family taxa associated with POTS.

Taxonomy	Regression coefficient	*P*-value	FDR	Median abundance (%)		Prevalence	
				Controls *N* = 39	POTS *N* = 32	Controls *N* = 39	POTS *N* = 32
Prevotellaceae	− 3.095	9.59e-04	0.021	0.132	7.30e-04	31	19
Acidaminococcace ae	− 2.369	0.004	0.047	0.034	0.00e + 00	30	11
Atopobioaceae	− 1.991	0.004	0.047	0.006	0.001	38	23
Pasteurellaceae	− 1.812	7.09e-04	0.021	0.003	0.00e + 00	31	11
Coriobacteriaceae	− 1.753	0.008	0.069	1.010	0.526	37	25

FDR = false discovery rate, POTS = Postural Orthostatic Tachycardia Syndrome. The median relative abundance (in %) of each taxon in each group is shown. Prevalence is the number of samples with a nonzero abundance for the given taxon. The effect size is represented as the coefficient for the group variable POTS in the linear regression model where the relative abundance is log transformed. Positive values imply the taxon is more abundant in the patients group compared to the controls. The FDR-adjusted *P*-value, adjusted for all tests, were performed within each taxonomic category and considered statistically significant if < 0.1. Redundant taxa have been omitted from the table.

**Figure 9:**
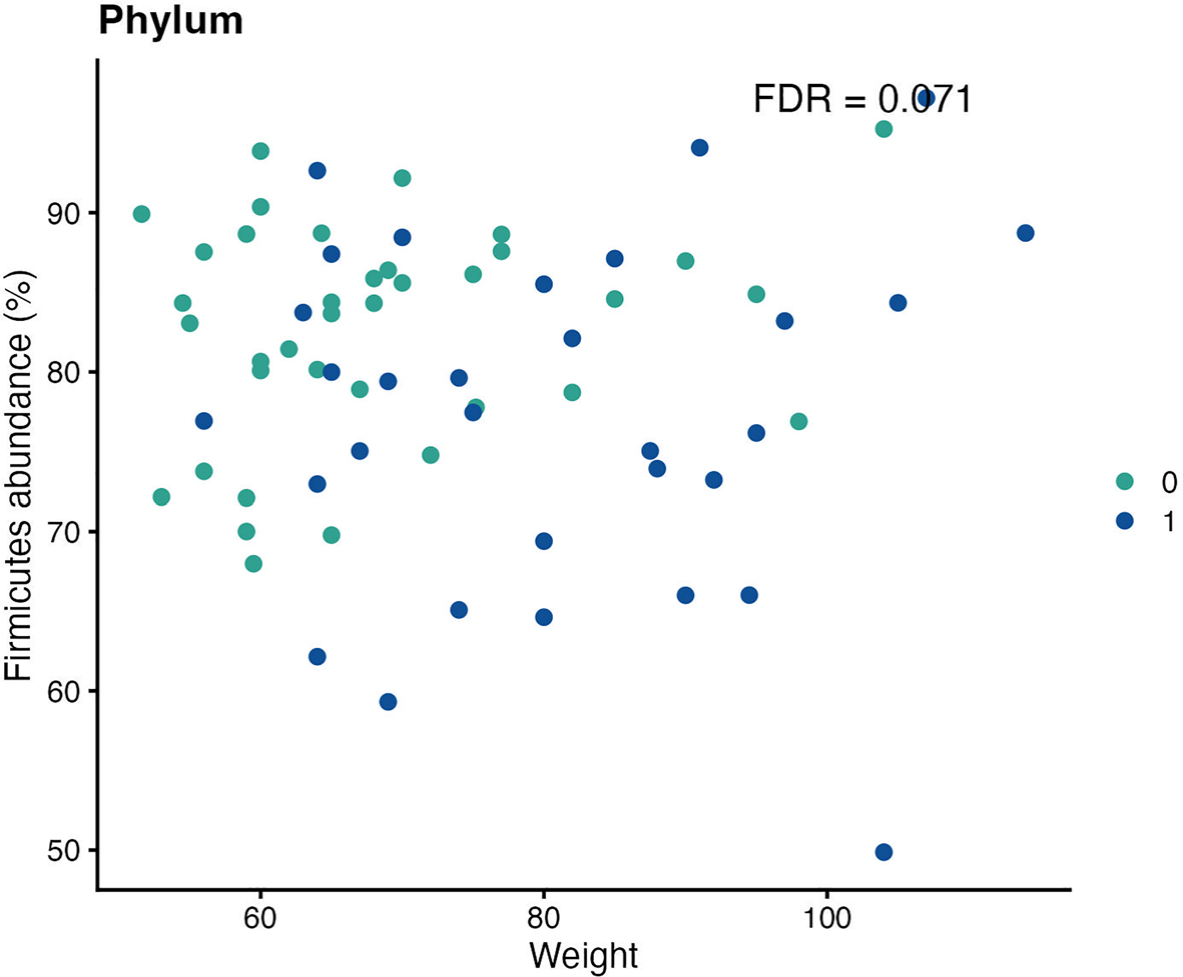
Scatter plot showing the confounding effect (weight) on the relative phylum abundance. Colours indicate the different groups (0 = controls, 1 = post-acute COVID-19 syndrome (PACS)). Even though individuals with PACS generally weighed more, there was still a significant difference in Firmicutes abundance between groups (False discovery rate (FDR) = 0.071).

The only significantly affected taxa for patients with PACS were the phyla *Ascomycota* and *Firmicutes* (Fig. 9) with effect sizes of—0.9077675 and -0.050807, respectively, and with FDR = 0.071 for both (Table 5 and supplementary file lundys_r1v4_COVID_regression_test_results.xlsx).

**Table 5:** Overview over regression analysis from the PACS group.

Category	Number of taxa	Number of significant taxa
MGS	733	0
Subspecies	18	0
Species	302	0
Genus	140	0
Family	43	0
Order	27	0
Class	18	0
Phylum	12	2
Superkingdom	3	0

PACS = Post-acute COVID-19 syndrome. A taxon was counted as significant if the false discovery rate (FDR)-adjusted *p*-value for the coefficient of the corresponding regression model was below 0.1. The FDR correction was done per taxonomic category.

### Correlation analysis

The total burden of gastrointestinal symptoms assessed as total IBS-SSS, fatigue, bloating and flatulence, were the clinical variables correlated with microbiome variables (taxonomic abundances, alpha diversity, MGS-level KEGG-module abundances, gene-level KO abundances, and GMM abundances) (supplementary file lundys_r1v4_correlation_wrapper_per-combination_order-by-FDR.xlsx).

Several items at the MGS, species, and genus levels correlated with total IBS-SSS, the specific symptom bloating and flatulence, and fatigue. Some correlations were also found among the other taxa. Alpha diversity measures were negatively correlated with all three clinical variables, with FDR < 0.1. Functional categories correlated with clinical variables, especially fatigue (Table 6). The overview table is also provided as a supplementary file lundys_r1v4_correlation_wrapper_overview_table.xlsx.

**Table 6:** Overview of correlated clinical variables with different microbiome variables.

Clinical variable	Category	Number of items passing prevalence filter	Number of significant items
IBS-SSS	MGS	670	106
IBS-SSS	Subspecies	17	0
IBS-SSS	Species	286	46
IBS-SSS	Genus	137	21
IBS-SSS	Family	42	0
IBS-SSS	Order	27	2
IBS-SSS	Class	17	1
IBS-SSS	Phylum	11	1
IBS-SSS	Superkingdom	3	0
IBS-SSS	Alpha diversity	2	2
IBS-SSS	KEGG module (MGS-level)	475	10
IBS-SSS	KEGG orthology (gene-level)	8075	200
IBS-SSS	GMM	101	17
Fatigue	MGS	679	208
Fatigue	Subspecies	17	0
Fatigue	Species	289	78
Fatigue	Genus	137	33
Fatigue	Family	42	2
Fatigue	Order	27	1
Fatigue	Class	17	2
Fatigue	Phylum	11	1
Fatigue	Superkingdom	3	0
Fatigue	Alpha diversity	2	2
Fatigue	KEGG module (MGS-level)	475	98
Fatigue	KEGG orthology (gene-level)	8498	905
Fatigue	GMM	101	32
Bloating	MGS	679	87
Bloating	Subspecies	17	0
Bloating	Species	289	33
Bloating	Genus	137	18
Bloating	Family	42	0
Bloating	Order	27	1
Bloating	Class	17	0
Bloating	Phylum	11	1
Bloating	Superkingdom	3	0
Bloating	Alpha diversity	2	2
Bloating	KEGG module (MGS-level)	475	24
Bloating	KEGG orthology (gene-level)	8498	116
Bloating	GMM	101	14

GMM = Gut Metabolic Modules, KEGG = Kyoto Encyclopedia of Genes and Genomes. Total IBS-Severity Scoring System (SSS) is calculated after Francis et al. (15). Specific gastrointestinal symptoms are assessed by the visual analog scale for IBS (VAS-IBS) (14). Symptoms are assessed on VAS scales 0–100 mm, where 0 means no symptoms and 100 maximal symptoms (15, 16). The correlation analysis was performed with Kendall’s method and with prevalence threshold of 10%.

## Discussion

The main findings of the present study were the lower richness in alpha diversity in patients with POTS and PACS compared with controls, and the differences in beta diversity between patient groups and controls. Furthermore, there were large differences in gut microbiota abundance between patients with POTS and controls at several taxa levels, whereas only the abundance of the phyla *Ascomycota* and *Firmicutes* differed between patients with PACS and controls. Gastrointestinal symptoms and fatigue correlated with alpha diversity and taxonomic and functional variables.

To compare microbiota composition from different studies is a great challenge since different studies use various methods and various designs. Furthermore, several diseases with gastrointestinal symptoms and fatigue have no objective signs of disease. Therefore, the diagnoses are based on symptom criteria, which are inconsistent over time^30^.

Only one previous study has examined fecal microbiota in POTS, which showed no significant differences in microbiota composition between POTS and controls^11^. The differences between the current and former studies may depend on different methods and fewer subjects in the former study^11^. Autonomic neuropathy leads to gastrointestinal dysmotility with subsequent alterations in microbiota composition, which may lead to bacterial overgrowth^31^. On the other hand, gut microbiota may contribute to the regulation of intestinal motility^32^. Thus, there is a bilateral direction of the interaction between gastrointestinal function and microbiota.

Animal models have shown that microbiota regulates the neuronal homeostasis and the important ratio between neurons and glial cells through involvement of toll-like receptors (TLR)^33^. Furthermore, some bacteria can produce neurotransmitters, e.g., nitric oxide (NO) and gamma-aminobutyric acid (GABA), which regulate intestinal transit time^34^. These interactions between microbiota and gastrointestinal motility and function may explain the correlations between gastrointestinal symptoms and bacteria composition, alpha diversity, and functional profiling in the current study.

Most human studies examining gastrointestinal dysmotility have been performed on diabetic patients. These studies are not completely comparable with POTS, since diabetes per se has great impact on metabolism and microbiota. However, clear differences between diabetes patients with and without autonomic neuropathy have been observed at all levels of the microbiota hierarchy^12^. One can assume that autonomic neuropathy with dysmotility should affect the body in a similar way, independent of genesis.

One of the most studied genera, *Lactobacillus*, has been shown to be associated with blood glucose levels, insulin resistance, and inflammatory biomarkers in diabetes^35^. The phyla *Bacteroidetes* and *Firmicutes* account for 90% of the total gut microbial composition, and *Bacteroidetes* is the most abundant Gram-negative gut bacteria^35^. *Bacteroidetes* exhibit anti-inflammatory properties, whereas the phylum *Firmicutes* is associated with obesity^29^. However, studies show inconclusive results, and no of these phyla were affected in the present POTS cohort. The taxa below phylum in the hierarchy, such as order, family, genus, and species exhibited several changes in abundances in POTS, but the changes were representative for all phyla, and the abundances for bacteria in lower hierarchies within the *Bacteroidetes* and *Firmicutes* phyla were both increased and decreased. Thus, the current study does not manifest these two phyla as the most important in autonomic neuropathy. Nevertheless, the phylum *Firmicutes* was one of two phyla with decreased abundance in patients with PACS.

Dietary habits have great impact on the microbiota composition^32^, and dietary interventions influence the gut microbiota^36^. There were only small differences in food intake between controls and patients in the current cohorts, with a higher intake of candies and cereals in controls than in patients. Therefore, dietary components could not explain the differences in composition between controls and POTS patients.

Chronic fatigue and lethargy are often observed in POTS and PACS conditions^4,37^. In parallel with autonomic dysfunction discussed above, stress and fatigue may alter both gastrointestinal secretion and motility through sympathetic and parasympathetic pathways, leading to altered microbiota^32^. Altered composition of gut microbiota may affect mood, sickness behavior, and fatigue trough inflammatory mechanisms involving gut epithelial permeability, the hypothalamic–pituitary–adrenal (HPA) axis, and the hippocampus^32^. Lethargy and chronic fatigue syndrome (CFS)/myalgic encephalomyelitis (ME) and their association with microbiota have not been frequently studied. In patients with autism, a correlation between lethargy/social withdrawal and beta diversity was observed^38^. Fatigue has in one study of CFS/ME been found to be associated with the abundance of *Actinobacteria*^39^.

The bidirectional crosstalk between the gut microbiota and the lungs, called the gut-lung axis, may explain the concomitant occurrence of fever, cough, fatigue, and gastrointestinal symptoms in COVID-19^40,41^. Several studies have suggested that dysbiosis is involved in COVID-19, and one study showed that patients with severe symptoms of COVID-19 had a greater proportion of the phyla *Campylobacterota* and *Actinobacteriota* than patients with milder symptoms^41^. Accordingly, probiotics have been shown to significantly improve symptoms of fatigue, breathlessness, and gastrointestinal complaints and shorten the disease duration^42^. The few abundance differences between patients with PACS and controls in the present study may be explained by the fact that most patients with PACS included had had a mild infection and had not required non-invasive or invasive ventilation. The frequent use of probiotics in the cohort with PACS may obscure the results.

One strength of the current study is the dietary registration, assuring similar food intake in both controls and patients from the same geographical area. The main limitation is the small size of the cohorts. Due to the size, only a few confounders could be considered without any possibilities to perform sensitivity analysis. For the current study we used an FDR of 0.1 according to the standard methodology at Clinical Microbiomics, Symbion. As can be seen in Tables 3–4, using a lower FDR would decrease the number of significant findings. The only significant association between controls and PACS, Ascomycota and Firmicutes, had an FDR of 0.071, thus using FDR of 0.05 or lower would attenuate the significance of this finding. All subjects were included from the same geographical area. Including a more diverse participant pool with various demographic and environmental factors with potential influence of gut microbiota would further increase the generalizability of study results. Sugar-rich soda was chosen since this was the only dietary intake that differed between POTS and controls in a larger study cohort, and the finding that POTS patients replaced meals by energy-rich drinks^13^. Accordingly, bloating was the most aggravated gastrointestinal symptom, and therefore calculated separately. The POTS patients were treated with several drugs and suffered from many other diseases in addition to POTS. Some of the differences observed in POTS may be caused by the drugs, which may obscure the pure disease effect. Nevertheless, the correlations between clinical characteristics and gut microbiota suggest a direct effect of the disease and not only by the drugs. Of the 39 control subjects with no signs of PACS or POTS, 15 had previously confirmed SARS-CoV-2. For 24 control subjects previous SARS-CoV-2 infection status was unknown. However, it is reasonable to assume that at the time of recruitment (March 2021-February 2022) at least some of these controls may have been in contract with SARS-CoV-2, accounting for the widespread prevalence of the infection in Sweden. Further, the cross-sectional design make causality impossible to determine. Thus, it is not possible to determine from the current study, whether the microbiota changes are due to the gastrointestinal symptoms, or also are related to other mechanisms. Longitudinal studies could provide more understanding of how microbial changes may affect symptoms over time. No information about antibiotic consumption among the participants or birth through caesarean section was available.

## Conclusions

Alpha diversity calculated as richness and Shannon was lower in the POTS cohort compared with the controls, whereas only richness was lower in patients with PACS compared with controls. Beta diversity differed between both patient groups and controls. Comparison of POTS and controls with linear regression adjusted for age, showed significant differences at MGS, species, genus, family, and order levels. When comparing patients with PACS and controls adjusted for weight, only two significantly affected phyla were found. Thus, the alterations of microbiota composition seem to be greater in patients with POTS than with PACS. Correlation analyses showed that the clinical variables total IBS-SSS, fatigue, and bloating and flatulence significantly correlated with multiple microbiome variables, such as alpha diversity, individual taxa abundances, and gene- or MGS-based functional group abundances.

### Supplementary Information

Supplementary Information 1.

Supplementary Information 2.

Supplementary Information 3.

Supplementary Information 4.

Supplementary Information 5.

Supplementary Information 6.

Supplementary Information 7.

Supplementary Information 8.

Supplementary Information 9.

Supplementary Information 10.

Supplementary Information 11.

## Data Availability

All data generated or analyzed during this study are included in this published article (and its Supplementary Information files).

## Abbreviations

BH: Benjamini–Hochberg
dbRDA: Distance-based redundancy analysis
FDR: False discovery rate
HQNH: High-quality non-host
KO: Kyoto encyclopedia of genes and genomes orthology
M: Million
MGS: Metagenomic species
PERMANOVA: Permutational multivariate analysis of variance
POTS: Postural orthostatic tachycardia syndrome
