# Supplementary material for: Gut microbiota composition is altered in postural orthostatic tachycardia syndrome and post-acute COVID-19 syndrome
Source: Sci Rep. 2024 Feb 9;14:3389. doi: 10.1038/s41598-024-53784-9 (PMC10858216; doi:10.1038/s41598-024-53784-9)

Supplementary Tables 1-2 and Supplementary Figures 1-3

Gut microbiota composition is altered in postural orthostatic tachycardia syndrome and post-acute COVID-19 syndrome

Viktor Hamrefors[1], Fredrik Kahn[2], Madlene Holmqvist[2], Katherine Carlson[2], Roosa Varjus[3], Alexander Gudjonsson[3], Artur Fedorowski[4], Bodil Ohlsson[5]

[1]Lund University, Department of Clinical Sciences; Skåne University Hospital, Department of Cardiology, Malmö, Sweden

[2] Lund University, Department of Clinical Sciences, Skåne University Hospital, Department of Infection Medicine, Lund, Sweden

[3]Clinical Microbiomics, Symbion, Copenhagen, Denmark

[4]Department of Cardiology, Karolinska University Hospital and Department of Medicine, Karolinska Institute, Stockholm, Sweden

[5]Lund University, Department of Clinical sciences; Skåne University Hospital, Department of Internal Medicine, Malmö, Sweden

Supplementary Table 1. Selected comorbidities and drug treatments

**Comorbidity**	**Recruited from POTS arm N = 27**	**Recruited from post-COVID-arm N = 32**
Asthma bronhialis	6	3
Myalgic Encephalomyelitis	4	N/A
Mast cell activation syndrome	3	N/A
Ehler-Danlos syndrome	3	N/A
**Drug treatment**		
Inhaled beta 2 agonists	5	1
Histamine H1 blockers	11	5
Histamine H2 blockers	6	0
Beta blockers	10	13
Antihypotensive drugs	9	1
Ivrabradine	9	3

POTS = Postural Orthostatic Tachycardia Syndrome. Patients from the post-COVID arm represent post-acute COVID-19 syndrome (PACS). The prevalence is given as absolute numbers.

Supplementary Table 2. Quality control and read mapping statistics

	**Minimum**	**Average**	**Maximum**
Read pairs	1.8 M	28.3 M	92.7 M
High quality reads	1.8 M	28.0 M	92.1 M
High quality non-host reads	1.8 M	27.8 M	92.1 M
Mapped to gene catalog	1.5 M	22.6 M	75.8 M
Gene catalog representation	76.3%	81.4%	84.0%

M = millions of reads. Average, minimum, and maximum values of quality control and read mapping statistics.

Supplementary Figure 1. Flow chart over patient recruitment

Supplementary Figure 1

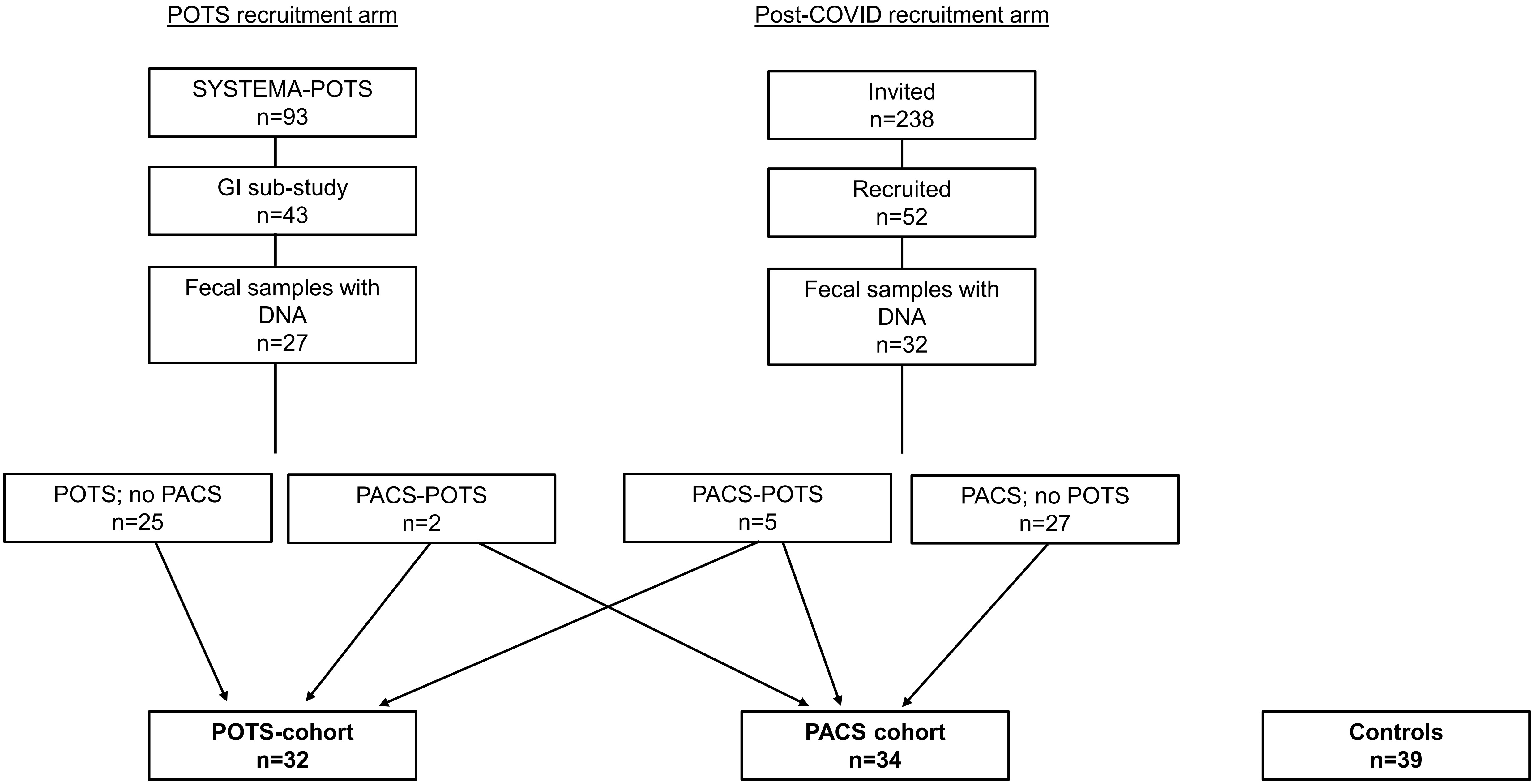

Supplementary Figure 2. Bar plots summarizing read quality and read mapping for all samples. Samples are ordered by increasing number of mapped reads.

Supplementary Figure 2

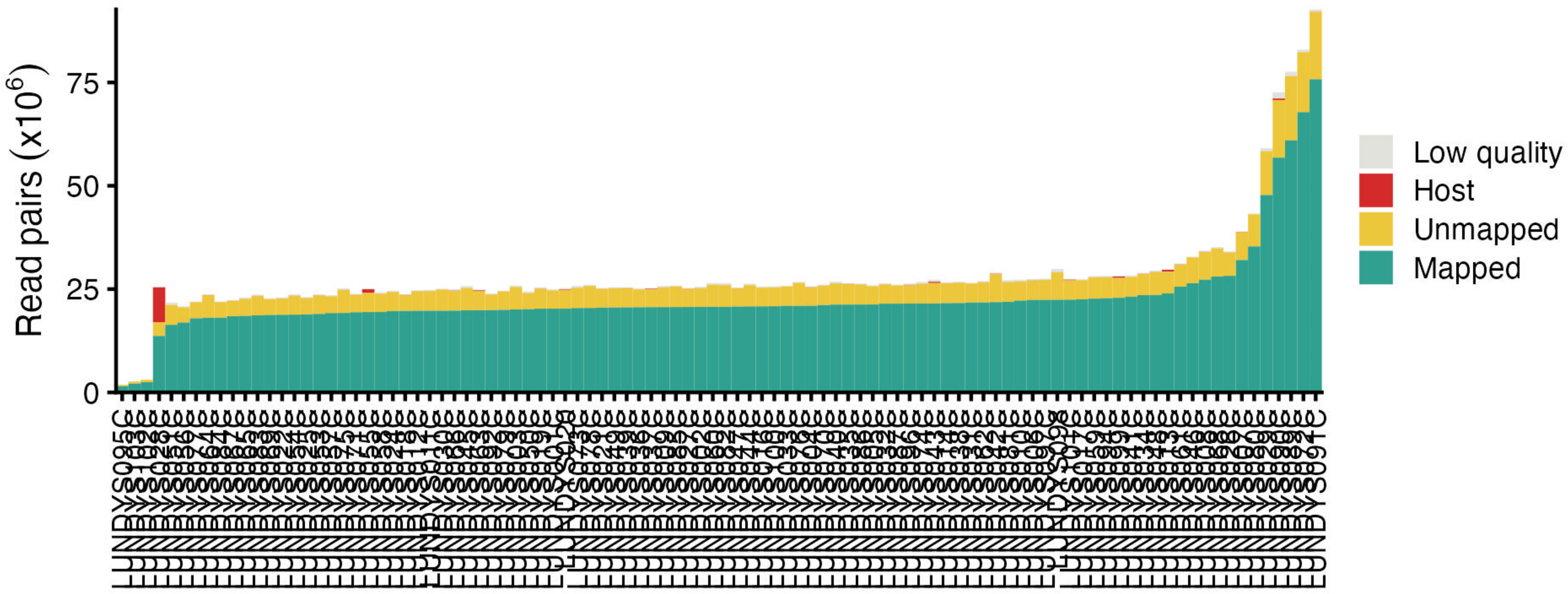

Supplementary Figure 3. Proportion of read pairs. Bar plots summarizing read quality and read mapping. Categories of different read types are given in percentages. Samples are ordered by increasing number of mapped reads.

Supplementary Figure 3

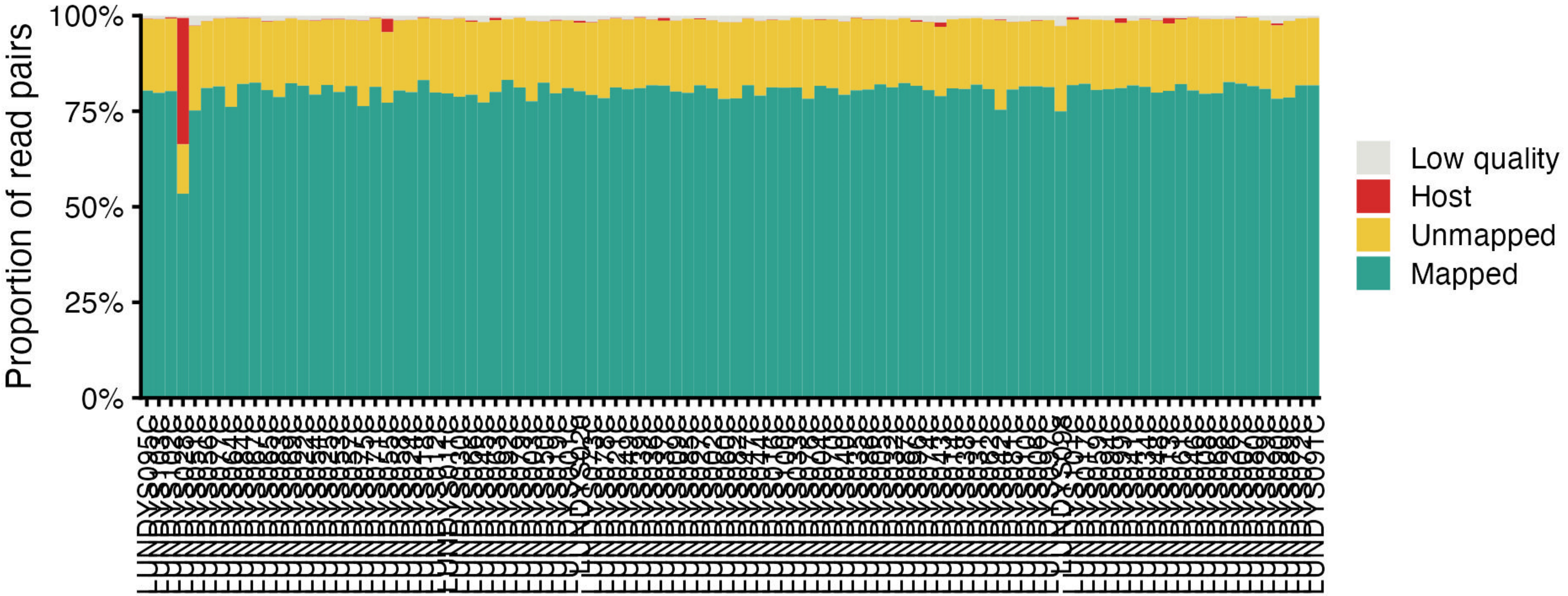

Supplement: Supplementary file 1 — Supplementary Information 1. [file 41598_2024_53784_MOESM1_ESM.pdf]
